# Efficacy of Aquatain, a Monomolecular Film, for the Control of Malaria Vectors in Rice Paddies

**DOI:** 10.1371/journal.pone.0021713

**Published:** 2011-06-29

**Authors:** Tullu Bukhari, Willem Takken, Andrew K. Githeko, Constantianus J. M. Koenraadt

**Affiliations:** 1 Laboratory of Entomology, Wageningen University and Research Centre, Wageningen, The Netherlands; 2 Kenya Medical Research Institute, Kisumu, Kenya; Université Pierre et Marie Curie, France

## Abstract

**Background:**

Rice paddies harbour a large variety of organisms including larvae of malaria mosquitoes. These paddies are challenging for mosquito control because their large size, slurry and vegetation make it difficult to effectively apply a control agent. Aquatain, a monomolecular surface film, can be considered a suitable mosquito control agent for such breeding habitats due to its physical properties. The properties allow Aquatain to self-spread over a water surface and affect multiple stages of the mosquito life cycle.

**Methodology/Principal Findings:**

A trial based on a pre-test/post-test control group design evaluated the potential of Aquatain as a mosquito control agent at Ahero rice irrigation scheme in Kenya. After Aquatain application at a dose of 2 ml/m^2^ on rice paddies, early stage anopheline larvae were reduced by 36%, and late stage anopheline larvae by 16%. However, even at a lower dose of 1 ml/m^2^ there was a 93.2% reduction in emergence of anopheline adults and 69.5% reduction in emergence of culicine adults. No pupation was observed in treated buckets that were part of a field bio-assay carried out parallel to the trial. Aquatain application saved nearly 1.7 L of water in six days from a water surface of 0.2 m^2^ under field conditions. Aquatain had no negative effect on rice plants as well as on a variety of non-target organisms, except backswimmers.

**Conclusions/Significance:**

We demonstrated that Aquatain is an effective agent for the control of anopheline and culicine mosquitoes in irrigated rice paddies. The agent reduced densities of aquatic larval stages and, more importantly, strongly impacted the emergence of adult mosquitoes. Aquatain also reduced water loss due to evaporation. No negative impacts were found on either abundance of non-target organisms, or growth and development of rice plants. Aquatain, therefore, appears a suitable mosquito control tool for use in rice agro-ecosystems.

## Introduction

Both urban and rural agriculture have been associated with increased risk of malaria to the local communities [1], [2]. However, the extent to which malaria transmission is affected by agriculture depends on many factors such as local climatic conditions, mosquito species, agricultural practices and economic conditions [1], [3]–[5]. In this regard, rice cultivation has attracted much attention as it provides abundant breeding opportunities for malaria mosquitoes. In addition, rice paddies are a challenging site for vector control [6]–[11]. The large size, slurry and vegetation make it difficult to effectively apply mosquito control agents in rice paddies and other, similar, habitats [12]. In our previous study we proposed Aquatain, a monomolecular film, as a suitable mosquito control agent for rice paddies based on its efficacy against *Anopheles* mosquitoes and its physical properties [13].

Monomolecular films differ from petroleum products due to their entirely physical and non-toxic mode of action [14], [15]. Monomolecular films act on mosquitoes by closing off their respiratory structures (siphons in larvae, trumpets in pupae) leading to suffocation [16]. Egg lecithin was the first monomolecular film to be rigorously tested in laboratory and rice paddies for its ability to control mosquito larvae [16]–[19]. It was followed by two ethoxylated isosteryl alcohol- based products, Arosurf® MSF (ISA-2OE or 66-E2) and Agnique® MMF [20]. These two products were tested against a variety of mosquito species in semi-permanent and permanent breeding sites, with and without vegetation, ranging from domestic water tanks, sewage treatment systems to salt marshes [21]–[29]. Different application methods and their effects on non-target organisms were evaluated [24], [28], [30]–[37]. Both products were also tested in combination with other mosquito larvicides such as *Bacillus thuringiensis* var. *israelensis*, *Bacillus sphaericus*, methoprene (an insect growth regulator), temephos and diesel oil [38]–[42]. The results showed that these monomolecular films could essentially be used for mosquito control provided they remained homogeneously spread over the treated site. However, these films not only had a tendency to accumulate around debris and vegetation, they also broke up by wind [20].

Aquatain (Aquatain products Pty Ltd., Australia), a new-generation product of monomolecular films, is silicone-based. It was originally designed as an anti-evaporation liquid and has the ability to self-spread over large water surfaces and around vegetation providing complete coverage of a large water body with emerging vegetation. The film formed by Aquatain is resilient to wind and rain [43]. It was found to be effective against the larval, pupal and adult stage of *Anopheles gambiae* Giles, *An*. *stephensi* Liston, *Aedes aegypti* L. and *Culex quinquefasciatus* Say in the laboratory [13], [44]. Aquatain (1 ml/m^2^) caused more than 90% mortality of *An. gambiae*, *An. stephensi* and *Cx. quinquefasciatus* larvae and 55% mortality of *Aedes aegypti* larvae. Pupae of all the species were extremely susceptible and 100% mortality was recorded within three hours. Aquatain treatment not only reduced the number of eggs deposited at the treated sites but also caused the ovipositing females to drown. Small-scale field trials were conducted in Sydney, Australia, which showed that Aquatain (1 ml/m^2^) reduced the densities of *Ae. notoscriptus* Skuse and *Cx. quinquefasciatus* larvae in buckets (0.30 m diameter), with and without plants, for six weeks after application [45].

The present study was carried out at the Ahero irrigation scheme in Kenya, where two monomolecular films, lecithin and Arosurf MSF, had been previously tested [18], [34]. The main objective of this study was to evaluate the efficacy of Aquatain as a mosquito control agent in rice paddies and investigate the potential side effects on non-target organisms and rice plants. Specific objectives were to (a) determine the impact of Aquatain treatment on larval and pupal densities of malaria vectors, (b) determine the impact of Aquatain treatment on emergence of adult malaria vectors, (c) evaluate the residual effect and retreatment interval for Aquatain, (d) determine the effect of Aquatain on non-target organisms, (e) determine the effect of Aquatain treatment on water evaporation, and (f) determine the effect of Aquatain treatment on the growth and development of rice plants.

## Materials and Methods

### Ethics statement

Scientific and ethical clearance was granted by the scientific steering committee and ethical review committee of the Kenya Medical Research Institute (SSC No. 1783-2^nd^ revision). A Memorandum of Understanding was signed with AIRS, which is a part of the National Irrigation Board (NIB), Kenya.

### Study area

**Figure 1:**
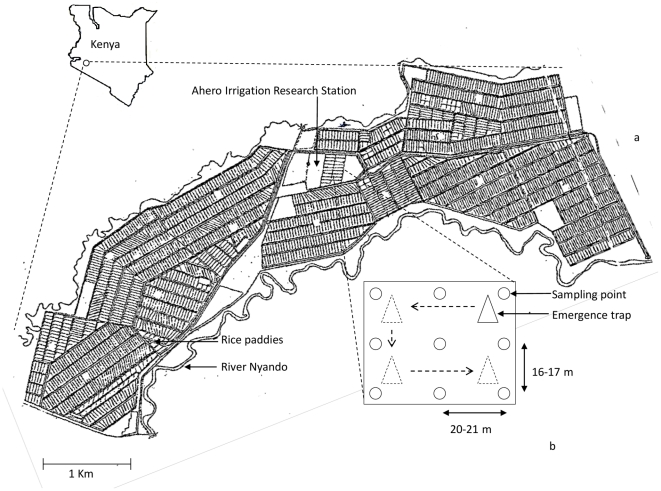
Ahero Irrigation scheme, sampling points for area sampler and emergence trap. Layout of rice paddies (1 acre each) in the Ahero Irrigation scheme (total area 2,168 acres) and location of the scheme in Kenya. The Ahero Irrigation Research Station (AIRS) is located within the scheme. The river Nyando is the water source for the scheme. a: Sketch of the entire scheme (courtesy of AIRS) and b: Schematic diagram showing the nine sampling points for the area sampler in a rice paddy, the emergence trap and the positions to which the emergence trap was moved.

Twelve 0.5 acre rice paddies (standard paddy size in Ahero: 1 acre) were selected within the Ahero Irrigation Research Station (AIRS, Figure 1a). The Ahero irrigation scheme (0°10'S, 34°55'E) is among three developed irrigation schemes in western Kenya. The main water source for this scheme is the river Nyando. The scheme irrigates 2,168 acres of farm land, most of which is used for rice cultivation. Presently there are 519 farming households connected to this scheme. The mean annual temperature ranges from 17°C to 32°C, the average annual rainfall from 1,000 to 1,800 mm and the average relative humidity is 65%. There are two rainy seasons: the long rains from March to August and the short rains from September to October [46]. This study was carried out during the long rains from March to June, 2010.

Malaria transmission occurs throughout the year in this area. *Anopheles gambiae* Giles *sensu stricto*, *An. arabiensis* Patton and *An. funestus* Giles are the main malaria vectors [46]. The mean annual *Plasmodium falciparum* Welch sporozoite inoculation rate (EIR) has been reported to be as high as 416 infective bites per person per year .

### Study design

A pre-test/post-test control group design was used for our field trial in the irrigated rice paddies. Baseline (pre-application) data were collected from all 12 experimental rice paddies for four weeks. Six paddies were then grouped as either control or treatment, so that on average the two groups matched in mosquito larval densities and adult emergence, the number of each non-target species caught, and rice plant characteristics (variety, height, plant density and tiller). Paddies were flooded before Aquatain application. The banks were then closed to prevent water from moving in or out of the paddies. Aquatain was then applied (first application) and data were collected for 19 days. The paddies were not supplied with additional water during this period. After 19 days the banks were opened to irrigate the paddies and then closed again before the second application of Aquatain. No further water was led in the following 20 days during which the data were collected.

### Rice crop

**Figure 2:**
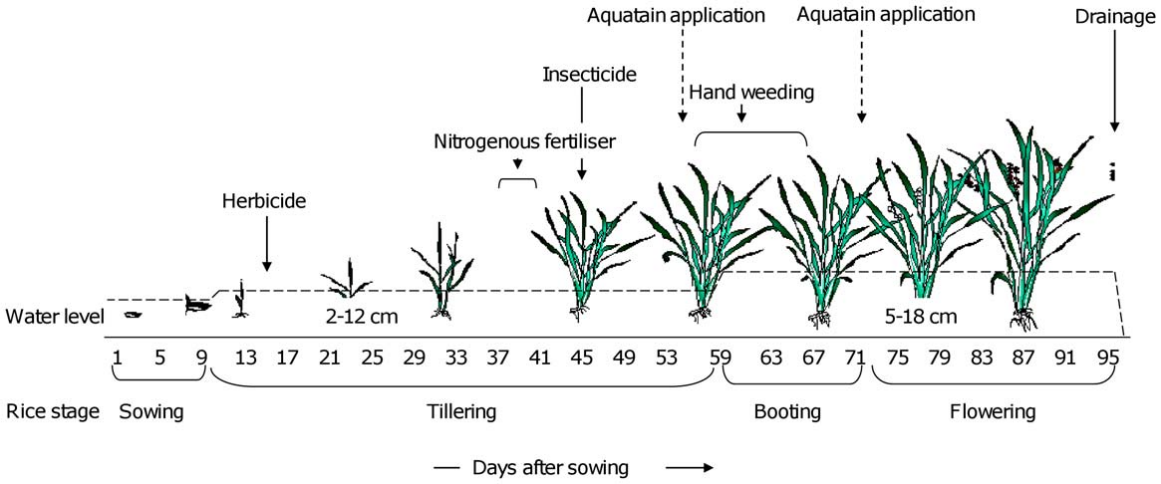
Timeline for rice growth, maintenance and application of Aquatain. The dashed horizontal line indicates the water level that was maintained during various stages of rice crop and drainage 10–14 days before harvest. The diagram also shows when a herbicide, nitrogenous fertiliser, and an insecticide were applied to the rice paddies. Broken arrows represent the two applications of Aquatain.

Sampling started 15–20 days after the rice seeds were sown (direct seeding) in the paddies (Figure 2). One of two available rice varieties, *Oryza sativa* ITA310 or *Oryza sativa* IR2793, was sown in each plot. After sowing, a thin water layer was maintained for 7–10 days to keep the soil moist. The water level was then increased to 2–12 cm for ∼45 days during the tillering stage. Then, the water level was increased to 5–18 cm for ∼30 days during the booting (obvious by stem swelling due to the panicle developing inside) and flowering (appearance of panicle) stage of the rice. The plots were drained 10–14 days before harvesting. Sampling ended when the plots were drained. During rice development, some plots were sprayed with a herbicide (Satunil 60 EC; Thiobencarb 40% and Propanil 20%) and a systemic insecticide (Titan, acetamiprid) as advised by AIRS. The insecticide is sprayed routinely for control of the rice stem borer, *Maliarpha separatella* Ragonot (Pyralidae: Lepidoptera). Insecticide spraying was also reported in the study by Reiter (1980) [18]. Apart from the insecticide spray a nitrogenous fertiliser was applied once in all the plots 5–6 weeks after sowing (Figure 2).

### Temperature, wind speed and rain fall measurements

The air temperature, wind speed, humidity and rainfall data were obtained from AIRS. The data were measured daily on-site.

### Larval sampling

Larval sampling was done every 4–5 days. In a laboratory study Aquatain caused >75% mortality after 5 days in both early (L_1–2_) and late (L_3–4_) stage larvae [13]. The first sampling was, therefore, done five days after Aquatain application. Larvae were collected with an area sampler, a bucket with the bottom cut out, by pushing the sampler until the lower rim touched the bottom of the field. A dipper or sweep net could not be used because the water level was only 2 cm during the early development stages of the rice plants. The area sampler had a height of 0.32 m with a diameter of 0.31 m at the top and 0.23 m at the bottom. The total area sampled was 0.085 m^2^. Nine sampling sites, along three transects, were selected and marked in each paddy (Figure 1b). Two transects were along the sides of the paddy while the third was made across the centre of the paddy. Each sampling point was 21 m apart from the other points in the same transect and 16–17 m apart from the corresponding point in the adjacent transect. The sampling site was not disturbed before the area sampler was quickly and firmly pushed into the soil. The contents captured in the sampler were removed with a plastic container and strained through a nylon cloth, separating the mosquito larvae, pupae and a variety of other (non-target) organisms from the water. The mosquito larvae and non-target organisms were placed in PET bottles with clean water and brought to the laboratory where the number of early stage (L_1–2_) larvae, late stage (L_3–4_) larvae and pupae of anopheline and culicine mosquitoes were counted. Late stage (L_3–4_) anopheline larvae were washed in hot water to clean and kill them for morphological identification. A sub-sample (30%) of *An. gambiae* s.l., kept in absolute alcohol, was analysed by PCR for identification to species level [47]. The pupae were reared till emergence and subsequently identified to genus level (*Culex* or *Anopheles*). These samples were not identified further.

### Adult sampling

**Figure 3:**
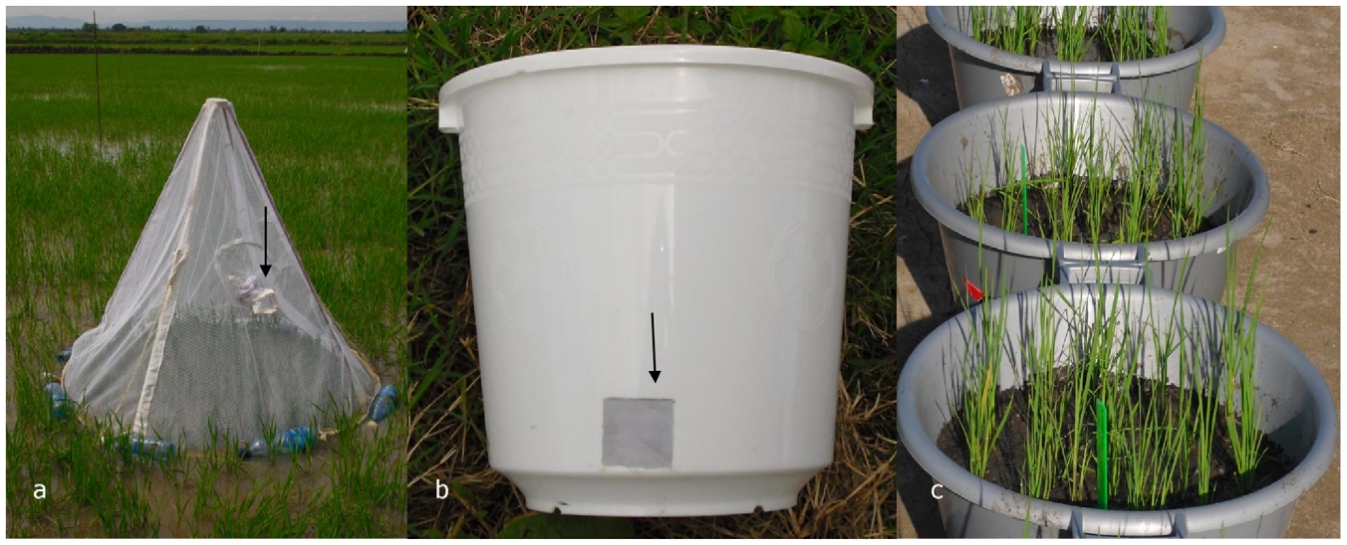
Emergence trap, field bioassay and evaporation measurement. a. Floating emergence trap. Arrow indicates the sleeve used to aspirate the adult mosquitoes and non-target organisms. b. Bucket used for the field bioassay on Aquatain efficacy. Arrow indicates the 25 cm^2^ holes at the bottom. c. Tubs with 15 rice plants, planted in a 20 cm soil layer. A 4 cm water layer was added to these tubs to measure evaporation.

As earlier laboratory studies suggested a quick reduction in adult emergence due to 100% pupal mortality within a few hours after exposure to Aquatain [13], [48], adult mosquitoes were collected daily from emergence traps (Figure 3a). The emergence trap consisted of a cone-shaped iron frame with a cover of mosquito netting. The cover was provided with a sleeve to allow easy aspiration of emerged adults [48]. The trap was 1 m high and had a diameter of 1 m at the lower side covering a surface area of 0.8 m^2^. PET bottles were used to keep the trap floating. This allowed free movement of larvae, pupae and non-target organisms, in and out of the area covered by the trap. The trap was tied to wooden stakes (inserted in the soil) to avoid being blown away or lifted by the wind. One trap was placed in each paddy. The position of all traps was changed on the same day in every paddy after four weeks (Figure 1b). Adult mosquitoes and non-target organisms found in the emergence traps were collected and brought to the laboratory where the numbers of *Culex* or *Anopheles* mosquitoes were recorded. *Anopheles* mosquitoes were morphologically identified to species level while *Anopheles gambiae* s.l. specimens were stored with silica gel and subsequently identified by PCR [47].

### Field bioassays

Field bioassays were carried out in the control and treatment paddies parallel to the first Aquatain application in the rice paddies. Plastic buckets (0.40 m high, 0.30 m diameter) with two 25 cm^2^ holes, 4 cm above the bottom, were placed in each paddy (Figure 3b). The holes were sealed with gauze which allowed free movement of water but prevented larvae from entering or leaving the bucket. The bucket was kept covered with a net. Forty second instar (L_2_) *An. gambiae s.s*. larvae (KEMRI strain, obtained from Kenya Medical Research Institute, Kisumu) were added to each bucket. The number of pupae that developed in each bucket was recorded daily. The pupae were then removed by a dipper.

### Aquatain treatment

Aquatain was poured in each paddy from a corner at the prescribed rate of 1 ml/m^2^ (1.4 L per paddy). The time taken for the Aquatain film to spread from one side of the paddy to the other was recorded. Aquatain was applied in the bioassay buckets, placed in the treatment paddies at a similar rate (1 ml/m^2^, 70 µl/bucket). Aquatain was applied separately inside the bioassay buckets because the water level was higher than the holes at the bottom preventing the Aquatain film from the paddy to enter the buckets. A second application of Aquatain (2 ml/m^2^, 2.8 L per paddy) was carried out at the moment the previously applied Aquatain film was no longer visible and the sampling showed similar larval densities in the control and treatment paddies.

### Water measurements

Water surface temperature, turbidity, presence of algae and depth were recorded daily in each paddy. Water surface temperature (5 mm top layer) was measured with a digital thermometer (GTH 175/Pt, Greisinger electronics, Germany). The water temperature was measured daily between 9:00–11:00. Water turbidity was categorized on a scale of 1 to 4, whereby ‘1’ referred to very clean water and ‘4’ to very turbid water. Presence or absence of algae was observed and recorded. In each paddy a steel rod (with a concrete base), firmly inserted into the soil, was used to measure the water level [10]. The distance between the water surface and the base was recorded daily.

### Evaporation measurements

Because the rice paddies were not leveled, the decrease or increase in water level in a paddy could not be translated into the volume of water added (by irrigation and rain) or lost (by evaporation or overflow). To establish whether Aquatain reduced the water loss by evaporation, bioassays were set up next to the rice paddies. Six plastic tubs (0.29 m high, 0.50 m diameter) were filled up to 20 cm with soil (Figure 3c). Fifteen rice seedlings (variety ITA-310) were planted in each tub. A nitrogenous fertiliser was added to these tubs while it was being applied in the paddies. Water was added to a level of 4 cm above the soil surface. Aquatain (1 ml/m^2^, 196 µl/tub) was applied to the water surface of three tubs. The water level was recorded every 2–4 days. After 12 days the same amount of Aquatain was re-applied.

In addition, assays were also carried out in similar tubs, without rice plants and soil, to measure the reduction in water loss by Aquatain treatment. In these assays six tubs were filled with water to the same level. Aquatain (1 ml/m^2^, 196 µl/tub) was applied to three tubs. After six days the volume of water required to refill each tub to the previous level was recorded. The assays were conducted twice with a total of six replicates.

### Non-target effects

#### a. Animals

Non-target organisms were collected from the area sampler and emergence traps to determine if Aquatain had any effect on their abundance. Non-target organisms were counted after dividing them into the following taxa; Ephemeroptera (mayfly nymphs and (pre-)adults), Odonata (damselflies, Zygoptera or dragonflies, Anisoptera nymphs and adults), Orthoptera (Gryllacrididae, crickets and Acrididae, grasshoppers), Diptera (Brachycera, house flies and biting flies), Heteroptera (bugs), Coleoptera (beetles), Lepidoptera (moths), Hymenoptera (Apocrita, wasps and Formicidae, ants), Arachnida (Hydrachnellae, water mite and Araneae, spiders), Molluscs (snails) Annelids (Hirudinea, leech and Haplotaxida, earthworms), fish (*Tilapia* or mudfish) and Amphibians (tadpoles and frogs). Heteropterans were further divided into Hydrometridae (water measurers), Veliidae (broad shouldered water striders), Gerridae (water striders) Nepidae (water scorpions), Corixidae (water boatmen) and Notonectidae (back swimmers) [48].

#### b. Rice plants

The average height and density of rice plants, average number of tillers per plant, and crop yield were recorded per paddy to be able to detect any negative effects of Aquatain on the growth and development of rice plants. The average height of the rice plant was recorded every two weeks. The height was the length from the bottom (soil) to the tip of the longest leaf blade. Three random measures were taken per paddy and the average was considered the representative of the whole paddy [10]. Plant density and number of tillers were measured every three weeks. Plant density was measured by counting the number of rice plants in a 1 m^2^ area. Number of tillers was measured by taking an average of the number of tillers in ten randomly selected plants per paddy [10]. The crop yield data was obtained from the AIRS after harvest.

### Statistical analysis

Larval density in the control and treatment paddies per sampling was compared by t-test after transforming the count data by log(x+1). Generalised estimating equations (GEE) were used to determine the difference in larval densities and adult emergence in the control and treated paddies adjusted for water level, water turbidity, water surface temperature, presence of algae, rice plant height and plant density. Larval densities and adult emergence were fitted to Poisson distribution by a logarithmic link function. First-order autoregressive relationship was used for the repeated measurements. Pre-application, first application and second application data were analyzed separately. Similarly GEE were used to detect differences in density of non-target organisms in the control and treated plots. In case of cyclops, however, presence or absence data was fitted to a binomial distribution by a logit link function before analysis by GEE.

Mulla's equation,
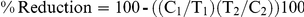
was used to calculate the percentage reduction in mosquito larval and pupal densities and adult emergence [49]. Where, e.g. in the case of larvae, C_1_ and T_1_ is the average number of larvae per sampling point in the control and treated paddies, respectively before Aquatain application. Similarly, C_2_ and T_2_ is the average number of larvae per sampling point in the control and treated paddies after Aquatain application.

Difference in water level and volume of water lost due to evaporation in the control and treated tubs was compared by t-test. Plant height, plant density, number of tillers and crop yield of the control and treated paddies were also compared by t-test. All analyses were performed using SPSS version 15 software (SPSS Inc. Chicago, IL, USA).

## Results

In total seven area sampler and 31 emergence trap collections were carried out during the 31-day base-line (pre-application) data collection. Control and treatment paddies (six each) were matched by mosquito larval densities and adult emergence, number of each non-target species caught, and rice plant characteristics (variety, height, plant density and tiller). The three exceptions were (pre) adult mayflies, water boatmen and water mites. The density of (pre) adult mayflies was lower in the control paddies (p<0.001, OR (odds ratio): 3.38, 95% confidence interval (CI): 1.53–3.80) compared to the treatment paddies. However, the density of water boatmen was higher in the control paddies compared to the treatment paddies (p<0.05, OR: 0.58, 95% CI: 0.37–0.90). Similarly the density of water mites was higher in the control paddies compared to the treatment paddies (p<0.05, OR : 0.56, 95% CI: 0.32–0.98). After the first Aquatain application (5 May 2010) three area sampler collections and 19 emergence trap collections were carried out during the 19-day data sampling. Extensive hand weeding was carried out during this period (Figure 2). Hand weeding involved a group of 8–10 female workers removing weeds from a rice paddy (0.5 acre) for 2–3 days (7–8 hrs per day). The film of Aquatain remained visible for 10 days. After the second Aquatain application (23 May 2010), three area sampler collections and 20 emergence trap collections were carried out during the 20-day data sampling.

### Temperature, wind speed, rainfall and relative humidity

**Figure 4:**
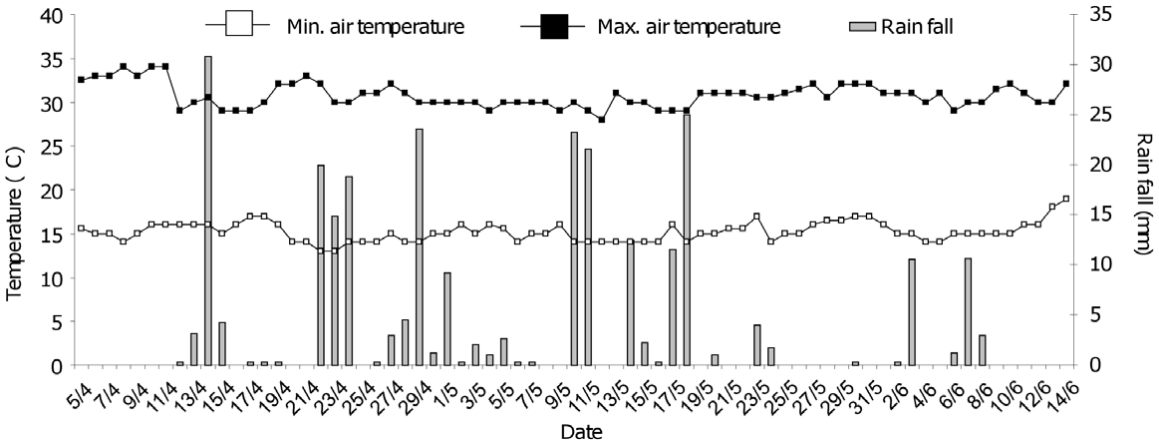
Air temperature and rainfall during the study period. Daily air temperature (minimum and maximum, °C) and rainfall (mm) measured during the study period (courtesy of AIRS).

The average minimum (± S.D.) and maximum (± S.D.) air temperatures during the study period were 15°C (±1.1) and 30.7°C (±1.3), respectively (Figure 4). Highest wind speed measured was 5.2 km/hr, five days after the first Aquatain application. Total rainfall was 270 mm over 71 days. The highest rainfall recorded before the first Aquatain application and after the first and second Aquatain application was 30.8 mm, 25 mm and 10.6 mm, respectively. The average relative humidity (± S.D.) was 56±13%.

### Larval sampling

Among the 3,071 mosquito larvae caught, 1,693 (55%) were anopheline and 1,378 (45%) were culicine. Late stage (L_3–4_) anopheline larvae (547) were morphologically identified as *An. gambiae* s.l. (40.9%), *An. pharoensis* Theobald (36%), *An. coustani* Laveran s.l. (14%), *An. squamosus* Theobald (1%), *An. ardensi* Theobald (0.9%) and *An. funestus* (0.1%). Some larvae (5%) could not be identified due to physical damage. A sub-sample of 67 *An. gambiae* s.l. larvae that was identified by PCR, consisted of 98.4% *An. arabiensis* and 1.5% *An. gambiae s.s*..

**Figure 5:**
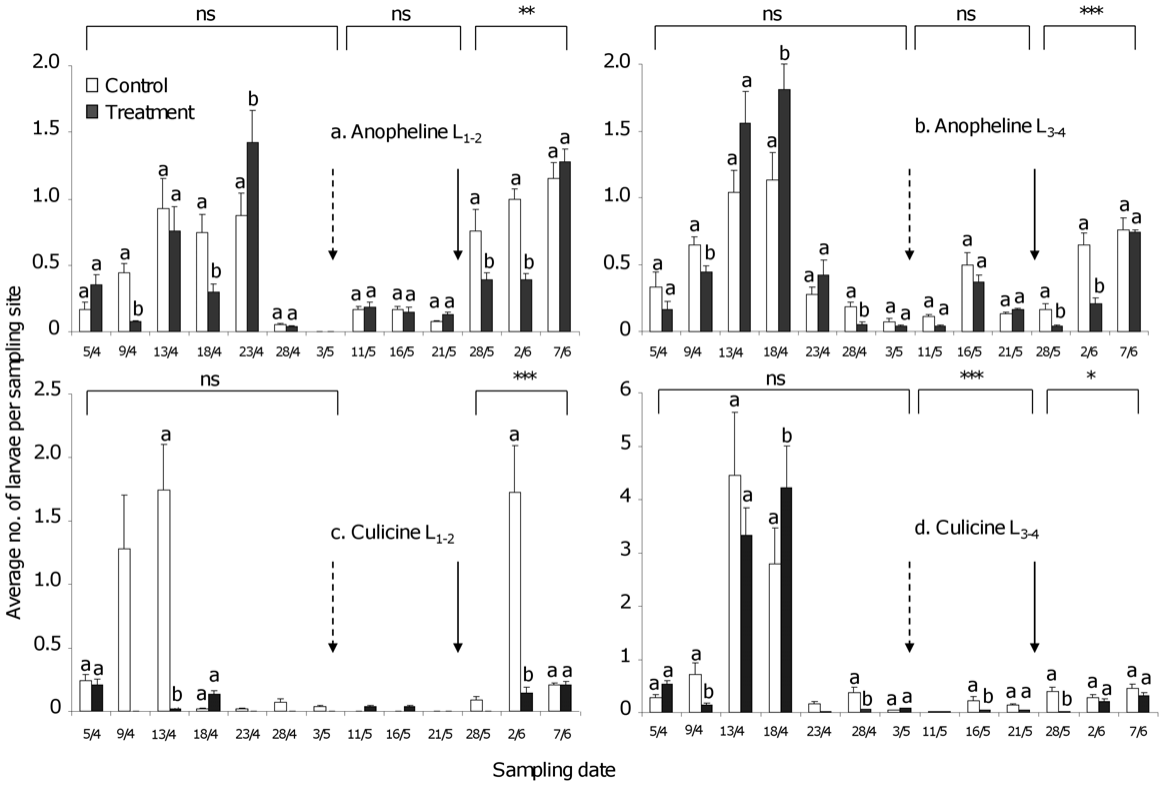
Average number (±S.E.) of early (L_1__–**2**_) and late (L_3–4_) anopheline and culicine larvae per sampling site. Average (± SE; n = 6) of early stage (L_1–2_) and late stage (L_3–4_) anopheline and culicine larvae per sampling point. The broken arrow indicates the first Aquatain application (1 ml/m^2^). The solid arrow represents the second Aquatain application (2 ml/m^2^). The square brackets on the top represent the overall comparison of the control and treated paddies before application (pre-application, 5/4–3/5) and after first (11/5–21/5) and second application (28/5–7/6). n.s., non-significant; *, p<0.05; **, p<0.01; ***, p<0.001 (Generalized estimating equations). Similar small case letters on every bar represent no significant difference (t-test, p<0.05) in the control and treatment paddies on that sampling date.

Overall, during pre-application samplings, larval densities in the paddies, later assigned as control or treatment, were similar (generalised estimating equations (GEE), p>0.05). Water turbidity had a negative effect on the number of young stage anopheline larvae (p<0.05, OR : 0.15, 95% CI: 0.03–0.71) and late stage culicine larvae (p<0.01, OR: 0.12, 95% CI: 0.02–0.58). However, anopheline and culicine larval densities were significantly different in paddies on various sampling dates (t-test, p<0.05, Figure 5).

Densities remained similar in the control and treatment paddies for young (p>0.05, OR: 1.0, 95% CI: 0.66–1.8) and late (p>0.05, OR: 0.6, 95% CI: 0.3–1.3) stage anopheline larvae after the first Aquatain application (1 ml/m^2^) (Figure 5a and 5b). For young stage culicine larvae, the data collected after the first Aquatain application could not be analysed because no larvae were found in the control paddies (Figure 5c). In the case of late stage culicine larvae, the overall larval density was lower (p<0.001, OR: 0.19, 95% CI: 0.12–0.32) in treatment paddies as compared to control paddies, but this effect was probably caused by the larval density being different (t-test, p<0.05) on one sampling date only (16 May 2010; Figure 5d). Water level in the paddies had a positive effect on the densities of late stage culicine larvae (p<0.01, OR: 1.05, 95% CI: 1.01–1.10). Larval density was higher when the water level was high. Similarly, the water surface temperature had a positive effect on the density of late stage culicine larvae (p<0.05, OR: 1.7, 95% CI: 1.07–2.7). Larval density increased with a higher water surface temperature. The water surface temperature (average ± S.D.: 24.5±2.17°C) ranged from 22°C to 31°C during this period.

Based on the results of the first Aquatain application, a double dose (2 ml/m^2^) of Aquatain was applied in the second application. Overall, larval densities were lower in the treatment paddies compared to the control paddies for young stage (p<0.01, OR: 0.6, 95% CI: 0.4–0.8) and late stage (p<0.001, OR: 0.2, 95% CI: 0.1–0.2) anopheline as well as young stage (p<0.001, OR: 0.07, 95% CI: 0.07–0.08) and late stage (p<0.05, OR: 0.2, 95% CI: 0.06–0.8) culicine larvae. Unlike after the first application, water level in the paddies had a negative effect on larval densities of young stage (p<0.001, OR: 0.92, 95% CI: 0.91–0.93) and late stage (p<0.05, OR: 0.94, 95% CI: 0.89–0.98) anopheline and young stage (p<0.001, OR: 0.72, 95% CI: 0.71–0.73) and late stage (p<0.05, OR: 0.9, 95% CI: 0.83–0.98) culicine larvae after the second Aquatain application. Larval densities decreased with an increase in water level. Water surface temperature had a positive effect on larval densities of young stage (p<0.001, OR: 2.2, 95% CI: 1.4–3.4) and late stage (p<0.001, OR: 4, 95% CI: 2.9–5.5) anopheline and young stage (p<0.001, OR: 11.1, 95% CI: 9.8–12.6) and late stage (p<0.05, OR: 6.2, 95% CI: 1.4–26.8) culicine larvae. Larval density was higher when the water surface temperature was higher. During this period the water surface temperature (average ± S.D.: 23.6±1.5°C) ranged between 20°C and 27.8°C. Turbidity had a negative effect on larval densities of late stage anopheline (p<0.001, OR: 0.49, 95% CI: 0.4–0.6) and culicine (p<0.05, OR: 0.1, 95% CI: 0.03–0.7) larvae. Larval density decreased when turbidity was high. Data collected after the second Aquatain application showed that larval densities were lower (t-test, p<0.05) in the treatment paddies compared to the control paddies for two consecutive collections (28 May 2010 and 2 June 2010) except for late stage culicine larvae where the difference was only observed for one collection (28 May 2010). The larval densities were no longer different in the control and treatment paddies during the third collection (7 June 2010).

Although there was no reduction in larval densities in the treatment paddies as compared to the control paddies after the first application, a 32% reduction in early stage anopheline and 19% reduction in late stage anopheline larvae was calculated using Mulla's equation. This equation also takes into account the larval densities in control and treatment paddies before application (Table 1). Anopheline larval densities were more reduced after the second application of Aquatain (Table 1). Considering the larval density after the first application, there was a reduction of 36% for early stage anopheline larvae and 16.4% for late stage anopheline larvae in the treatment paddies after the second application (Table 1). Reduction could not be calculated for young culicine larvae because no larvae were caught in the treatment paddies before the first Aquatain application and in the control paddies after the second Aquatain application (Table 1). There was no reduction in case of late stage culicine larvae.

**Table 1:** Average number (S.E.) of larvae, pupae and adults collected before and after Aquatain applications.

Mosquito	Stage	Pre-application		First application		Second application		% Reduction compared to		
								pre-application after		first application after
		Control	Treatment	Control	Treatment	Control	Treatment	first	second	second
								application		
*Anopheles*	L_1–2_	0.31 (1.80)	0.49 (2.60)	0.97 (1.80)	0.69 (1.30)	0.14 (0.50)	0.15 (0.60)	32.2	55.0	36.0
	L_3–4_	0.18 (0.80)	0.17 (0.90)	0.52 (1.09)	0.33 (0.79)	0.25 (1.02)	0.19 (0.54)	19.5	32.8	16.4
	Adult	0.20 (0.05)	0.22 (0.09)	0.51 (0.11)	0.07 (0.04)	0.65 (0.11)	0.05 (0.02)	93.2	87.5	0
*Culex*	L_1–2_	0.04 (0.28)	0	0.70 (0.58)	0.12 (0.60)	0	0.02 (0.20)	a	a	a
	L_3–4_	0.19 (1.05)	0.04 (0.24)	0.37 (1.15)	0.17 (0.69)	0.12 (1.01)	0.03 (0.02)	3	0^b^	0^b^
	Adult	0.39 (0.06)	0.34 (0.04)	1.03 (0.18)	0.17 (0.06)	0.35 (0.07)	0.09 (0.04)	69.5	81.5	39.3
Both	Pupal	0.03 (0.01)	0.04 (0.01)	0.02 (0.01)	0.01 (0.01)	0.09 (0.04)	0.04 (0.02)	60.8	76.5	40.0

a.Not calculated.

b.Larval densities increased over previous (pre-) application.

Average number of larvae (±SE) and pupae (±S.E.) per sampling point per sampling day and average number of adults (±S.E.) per emergence trap per day collected before (Pre-application) and after applying 1 ml/m^2^ (first application) and 2 ml/m^2^ (second application) of Aquatain. Percentage reduction after first and second application compared to pre-application and after second application compared to first application are given.

In total, 53 pupae were collected. Among these only 10 yielded anopheline mosquitoes. Five pupae were caught after the first Aquatain application (three in control paddies and two in treatment paddies). A 60% reduction in pupae was calculated using Mulla's equation (Table 1). Twenty one pupae were caught after the second Aquatain application (15 in control paddies and six in treatment paddies). A 76% reduction in pupae was calculated by Mulla's equation when compared to the number of pupae before the first Aquatain application and 40% when compared to the number after the first Aquatain application (Table 1). The total number of pupae in the treatment and control paddies before the first application and after the first and second application was too small for statistical comparison.

### Adult sampling

Among the 549 mosquito adults caught from emergence traps, 222 (40%) were anopheline and 327 (60%) were culicine. Anophelines were morphologically identified to be *An. coustani* s.l. (39%), *An. pharoensis* (29%), *An. gambiae* s.l. (11.4%), *An. ziemanni* Grunberg (6.8%) and *An. funestus* (0.4%). Some adults (6.8%) could not be identified due to damaged body parts. All *An. gambiae* s.l. (25) specimens were identified by PCR and consisted of *An. arabiensis* (76 %) and *An. gambiae s.s*. (24%). The difference in the ratio of *An. gambiae s.s*. and *An. arabiensis* between the larval and adults stage was significant (χ^2^ = 13.1; p<0.001; df = 1).

**Figure 6:**
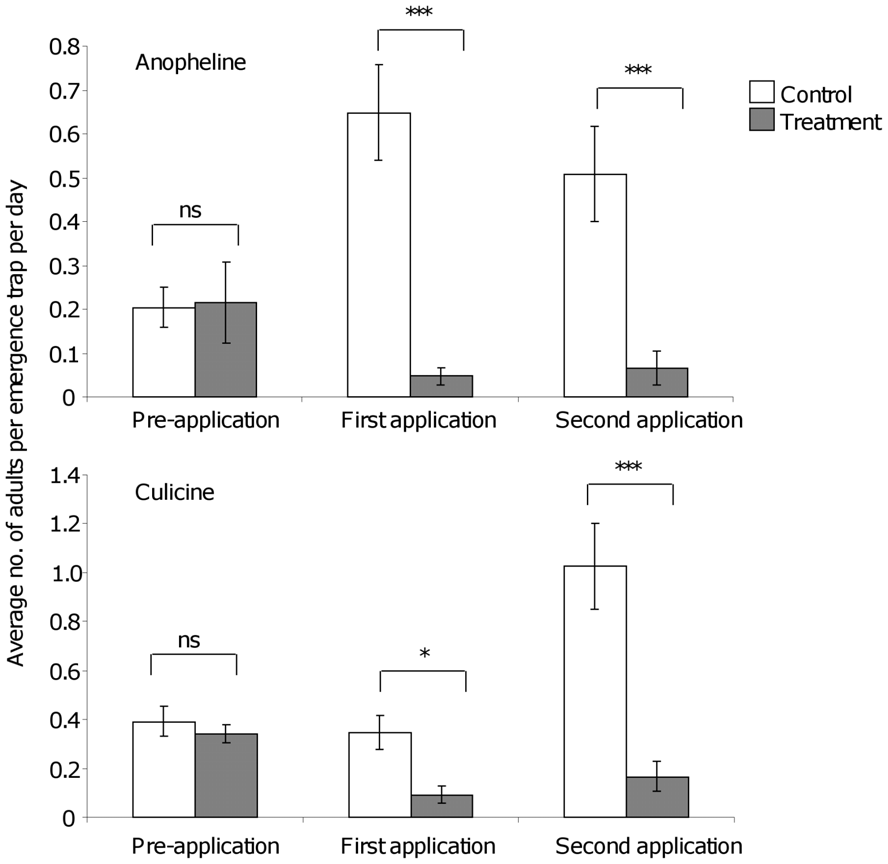
Average number of anopheline and culicine adults in the emergence trap. Average number (± SE; n = 6) of anopheline and culicine adults collected in the emergence trap per day in the control and treatment paddies before application (pre-application) and after first and second application. n.s., non-significant; *, p<0.05; ***, p<0.001 (Generalized estimating equations).

During pre-application collections, there was no difference in anopheline (p>0.05, OR: 1.05, 95% CI: 0.4–2.7) and culicine (p>0.05, OR: 0.7, 95% CI: 0.3–1.5) adult emergence in paddies, that were later assigned as control and treatment paddies (Figure 6). However, after the first Aquatain application (1 ml/m^2^), adult emergence was significantly lower in the treatment paddies compared to the control paddies for both anophelines (p<0.001, OR: 0.07, 95% CI: 0.02–0.18) and culicines (p<0.05, OR: 0.24, 95% CI: 0.07–0.75). Similarly, after the second application of Aquatain (2 ml/m^2^), the adult emergence remained lower in the treatment paddies compared to the control paddies for both anophelines (p<0.001, OR: 0.12, 95% CI: 0.04–0.35) and culicines (p<0.001, OR: 0.16, 95% CI: 0.08–0.3). During pre-application collections turbidity had a negative impact on culicine adult emergence (p<0.01, OR: 0.3, 95% CI: 0.2–0.6). Fewer adults emerged when water turbidity was higher.

The first application of Aquatain caused a 93% reduction in the emergence of anopheline adults and 70% reduction of culicine adults (Table 1) considering the emergence in the control and treatment paddies before treatment (pre-application). Similarly, there was an 88% reduction in emergence of anopheline adults and 82% reduction of culicine adults after the second Aquatain application (Table 1). There was a reduction of 40% in culicine adult emergence but no additional reduction in anopheline adults after the second application compared to the adult emergence after the first application. Among the 17 *An. gambiae* s.l. adults collected after Aquatain application only one (6%) originated from the treated paddies.

### Field bioassays

No pupation was recorded from *An. gambiae s.s*. larvae added to the Aquatain-treated buckets in the paddies, while in the control buckets 41±11% of the larvae pupated.

### Aquatain treatment

It took an average of 26 (±2) minutes for the Aquatain to spread across the rice paddy. The spreading was influenced by the direction of the wind. Aquatain spread relatively faster when the wind direction was with the side from where the Aquatain was placed on the water.

### Evaporation measurements

**Figure 7:**
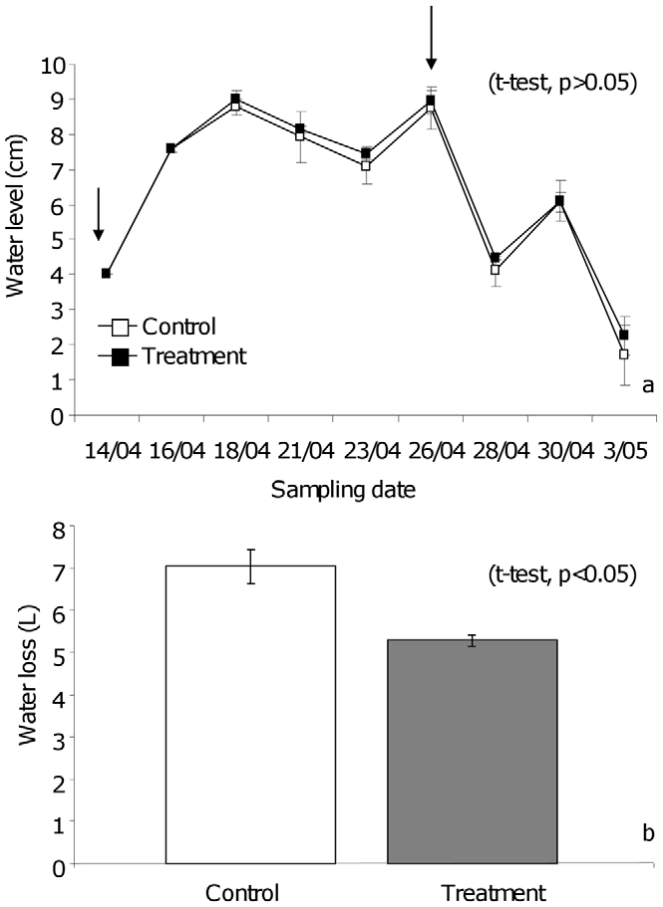
Water level and volume as a measure of water loss due to evaporation. a: The water level (cm) in the control and treated tubs (with rice plants and soil) on each sampling date. Arrows represent Aquatain application (1 ml/m^2^). b: The volume of water lost (Liters) from the control and treatment tubs in the assays (without rice plants and soil).

The water level was not significantly different (t-test, p>0.05) between the tubs that contained soil and rice plants and were treated with either Aquatain or were left untreated (Figure 7a). The observed increase in water level in the tubs during the sampling period (Figure 7a) is due to either rainfall or watering. In the assay without soil and plants however, nearly 1.7 liters more water (t-test, p<0.05, Figure 7b) was required to top up the control tubs (7.03±0.34 L) as compared to the treated tubs (5.28±0.14 L). During these days the average minimum and maximum air temperature were 15.2°C and 30.5°C, the average relative humidity was 55% and total rainfall was 14.8 mm.

### Non-target effects

#### a. Animals

There was no difference (GEE, p>0.05) in the densities of non-target organisms in the control and treatment paddies after Aquatain application apart from backswimmers (Table 2). After the second Aquatain application, fewer backswimmers were found in the treatment paddies compared to the control paddies (p<0.01, OR: 0.3, 95% CI: 0.1–0.7). After the first Aquatain application the densities of tadpoles and Mayfly (pre-) adults were lower in the control paddies compared to the treatment paddies (Table 2). Cyclops, taken into account as present or absent per sampling point, showed no significant difference before or after first and second Aquatain application (p>0.05). In case of some organisms, too few individuals were caught over the entire study period for analysis. These are adult dragonflies (8), crickets (6), grass hoppers (21), water measurers (27), water striders (3), moths (33), ants (3) and spiders (19). In general, turbidity had a negative effect on the densities of many non-target organisms.

**Table 2:** Average number ± S.E. (total number) of non-target organisms collected with the area sampler or emergence traps.

Taxon	Organism	Pre-application		First-application		Second-application	
		Control	Treatment	Control	Treatment	Control	Treatment
Ephemeroptera	Mayfly nymphs	1.08±0.18 (523)	1.33±0.18 (501)	0.98±0.16 (159)	1.28±0.17 (207)	1.30±0.21 (216)	1.15±0.17 (187)
	Mayfly (pre-) adults	0.94±0.19 (174)	2.25±0.33 (418)***	0.10±0.04 (11)	0.77±0.16 (83) ***	0.27±0.04 (30)	0.27±0.11 (34)
Odonata							
*Zygoptera*	Damselfly nymphs	0.06±0.01 (28)	0.04±0.02 (20)	0.42±0.13 (68)	0.35±0.06 (56)	0.73±0.15 (115)	0.60±0.09 (97)
	Damselfly adults	0.20±0.06 (76)	0.17±0.49 (68)	0.03±0.16 (3)	0.02±0.13 (2)a	0.24±0.06 (39)	0.34±0.14 (56)
*Anisoptera*	Dragonfly nymphs	0.15±0.03 (55)	0.16±0.02 (60)	0.18±0.04 (29)	0.22±0.037 (35)	0.44±0.06 (68)	0.57±0.08 (93)
Heteroptera							
*Veliidae*	Broad shouldered water strider	0.19±0.04 (73)	0.13±0.02 (49)	0.62±0.12 (100)	0.46±0.22 (75)	0.04±0.02 (7)	0.01±0.01 (1)a
*Nepidae*	Water scorpion	0.01±0.02 (5)	0.04±0.02 (8)	0.07±0.02 (12)	0.06±0.02 (10)	0.06±0.20 (9)	0.06±0.35 (10)
*Corixidae*	Water boatmen	1.32±0.21 (500)	0.59±0.93 (223)*	1.31±0.12 (213)	1.20±0.11 (195)	0.02±0.01 (3)	0.01±0.01 (2)a
*Notonectidae*	Back swimmers	0.59±0.76 (222)	0.79±0.11 (300)	0.83±0.11 (135)	0.79±0.13 (128)	0.77±0.15 (124)	0.30±0.05 (49)**
Coleoptera	Water beetles	13.89±1.32 (5187)	13.99±1.89 (5279)	3.43±0.46 (556)	3.76± 0.23 (610)	0.01± 0.01 (2)	0.03±0.01(5)a
Diptera							
*Brachycera*	House flies, biting flies	0.16±0.03 (30)	0.15±0.03 (27)	0.01±0.01 (1)	0.06±0.01 (6)a	0.03±0.01 (3)	0 (0)a
Hymenoptera							
*Apocrita*	Wasp	0.02±0.01 (4)	0.01±0.009 (1)a	0.93±0.12 (151)	0.85±0.11 (138)	0.01±0.01 (2)	0.04±0.01 (7)a
Arachnida							
*Hydrachnellae*	Water mite	0.25±0.03 (93)	0.13±0.02 (51)*	0.07±0.02 (12)	0.11±0.70 (18)	0.01±0.01 (2)	0.02±0.01 (4)a
Molluscs	Snails	0.21±0.04 (57)	0.29±0.04 (87)	0.03±0.01 (3)	0.04±0.01 (4)a	0.12±0.69 (21)	0.14±0.01 (23)
Annelids							
*Hirudinea*	Leech	0.01±0.01 (1)	0.01±0.01 (1)a	0.10±0.12 (17)	0.12±0.11 (20)	0.01±0.01 (2)	0.01±0.01 (2)a
Fish	Tilapia, mudfish	0.01±0.01 (5)	0 (0) a	0.14±0.10 (22)	0.06±0.02 (10)	0.25±0.06 (41)	0.19**±**0.07 (30)
Amphibian	Tadpoles	0.69**±**0.26 (261)	0.45**±**0.06 (171)	0.75**±**0.16 (121)	1.41**±**0.20 (229) **	0.54**±**0.09 (88)	0.67**±**0.11 (109)
	Frogs	0.03**±**0.02 (7)	0.01**±**0.05 (3)	0.06**±**0.03 (11)	0.03**±**0.01 (6)	0.02**±**0.01 (2)	0 (0)a

a.sample size too small for analysis.

Average number **±** S.E. (total number) of non-target organisms collected with the area sampler or emergence traps before and after the first and second Aquatain application. The numbers of each non-target organism in the control and treatment paddies, before and after the first and second Aquatain application, was compared by generalized estimating equations (n.s., no significant difference; *, p<0.05; **, p<0.01; ***, p<0.001).

#### b. Rice plants

There was no difference (t-test, p>0.05) in the average plant height, plant density, average number of tillers per plant in the control and treatment paddies after Aquatain applications (Table 3). The crop yield from the control and treatment paddies was also similar (t-test, p>0.05, Table 3).

**Table 3:** Average (S.D.) Plant height, plant density, number of tillers and crop yield in the control and treatment paddies.

Characteristic	Application								
	Pre-			First			Second		
	Control	Treatment	p-value	Control	Treatment	p-value	Control	Treatment	p-value
Plant height	39.5 (2.2)	39.5 (2.2)	0.97	67.9 (1.3)	66.8 (1.7)	0.61	78.5 (1.1)	77.5 (1.0)	0.53
Plant density	278 (31.4)	301 (25)	0.72	267 (43.1)	263 (21.3)	0.29	270 (42)	265 (22.2)	0.26
Tiller	6.6 (1.1)	7.2 (1.2)	0.56	12.9 (1.6)	15.5 (1.8)	0.93	13.3 (1.4)	15.7 (1.5)	0.93
Yield	-	-	-	-	-	-	984 (26)	958 (20)	0.45

Average (S.D.) Plant height (cm), plant density (plants/m^2^) and number of tillers (per plant) in the control and treatment paddies before and after the first and second Aquatain application. The average crop yield (kg/plot) of the control and treatment paddies is also given. n.s., no significant difference (t-test, p>0.05).

## Discussion

This study shows that Aquatain can significantly reduce larval densities and adult emergence of both anopheline and culicine mosquitoes in rice paddies without affecting other aquatic non-target organisms. Moreover, Aquatain has no negative impact on the rice crop and has a potential to save water, under climatic conditions in Kenya, required for the irrigation of rice.

Of all larvae collected, *Anopheles gambiae* s.l. was the most abundant anopheline species. In adult mosquitoes, however, *An. gambiae* s.l. (11.4%) was predominated by *An. coustani* s.l. (39%) and *An. pharoensis* (29%). Among *An. gambiae* s.l, the ratio of *An. gambiae s.s*. and *An. arabiensis* varied in the larvae and adults. Compared to the larval stage, relatively more *An. gambiae s.s* were found in the adults stage. This indicates a higher survival of *An. gambiae s.s*. larvae as compared to *An. arabiensis* larvae. Paaijmans *et al*. (2009) in field bioassays showed that in a mixed population with high proportion of *An. arabiensis* larvae, the total development time of *An. gambiae s.s*. larvae was 0.6 day shorter compared to development time in a single species population. Reduced development time reduces threats of predation, pathogens and unfavorable climatic conditions. Apart from the reduced development time, the mortality of *An. arabiensis* was also found to be higher than *An. gambiae s.s*. [50]. This might have led to the higher proportion of *An. gambiae s.s*. in the adults. However, overall *An. arabiensis* were more abundant. *Anopheles arabiensis* is known to be the most abundant species in the study area and Kenyan rice lands in general [3], [34], [51]. *Anopheles gambiae s.s.* has not been previously reported from Ahero [18], [34]. However, this study was carried out after the El Nino rains (that occurred in the last quarter of 2009 and continuing into the first quarter of 2010). These rains presumably cause high humidity conditions suitable for invasion and survival of *An. gambiae s.s.* [52]. The proportion of *An. gambiae s.s*. larvae and adults in a mixed population of *An. gambiae s.s*. and *An. arabiensis* was reported to be higher when humidity conditions were high in Mivani, a small rural village (0°06S, 34°45E) situated 14 km northeast of the Ahero irrigation scheme [53].

Aquatain effectively reduced the number of adult mosquito emergence, which is one of the factors that can directly determine malaria prevalence [34], [54]. In Aquatain-treated plots a high reduction in the emergence of both anopheline (93%) and culicine (69 %) adults was observed. Karanja et al. (1994) and Ali (2000) reported similar results for the adult emergence of anopheline and chironomid midges, respectively [21], [34]. The emergence of adults from the pupal-case relies heavily on the surface tension of the water, which is reduced by Aquatain and other monomolecular films [19]. Reduced surface tension not only prevents adults from emerging but also from ovipositing [19].

Among the immature stages of mosquitoes, pupae are most sensitive to monomolecular films, followed by late stage (L_3–4_) and early stage (L_1–2_ ) larvae [13], [22], [23]. Our study found similar results for pupae with a 60% reduction after the first Aquatain application. However, in the case of early and late stage larvae, a greater reduction was observed in the early stage larvae (36%) compared to the late stage ones (16.4%) after the second Aquatain application. A possible explanation for this difference might be that the first larval collection was carried out five days after Aquatain application so the late stage larvae caught were actually those larvae that were in an early stage (L_1–2_) when Aquatain was applied, and hence less affected. The larvae that were in a late stage when Aquatain was applied, pupated and emerged when in the control paddies and were killed when in the treated paddies. So the actual effect of Aquatain application on late stage larvae could not be observed. The higher reduction observed in the case of early stage larvae maybe due to reduced oviposition because the Aquatain film, as mentioned above, causes ovipositing female mosquitoes to drown [13].

Larvae, in contrast to pupae and adults, are less affected by the reduced surface tension because of their ability to utilise dissolved oxygen, which makes it possible for them to avoid the water surface for a certain period of time [16]. The concentration of dissolved oxygen, therefore, has a very important role in determining the efficacy of a monomolecular film. In the field, there were many factors that could influence the dissolved oxygen concentration. Factors that could decrease the concentration of dissolved oxygen are high water turbidity and low temperature [17], [18], [24]. Irrigation and rainfall, on the other hand, may have increased the concentration of dissolved oxygen [18]. During day time, the concentration of dissolved oxygen may have increased because of high temperatures and release of oxygen from photosynthesis in the algae [18]. The presence of algae in the control and treatment paddies might, therefore, be a contributing factor to the less harmful effect of Aquatain on larvae compared to that observed in the laboratory studies [13], [44]. The small pockets of untreated water surfaces formed in between the thread-like mass of algae, where Aquatain did not reach, might have also contributed to the less harmful effect on larvae.

Another explanation of why Aquatain has less effect on larvae compared to pupae and emerging adults is that a lower surface tension is required to disrupt larval breathing as compared to pupal breathing or adult emergence. Aquatain reduces the surface tension of water surface to 21.2 dynes/cm at 25°C. Normally at this temperature, the water surface tension is 71 dynes/cm. A surface tension that is reduced to as much as 27–36 dynes/cm can prevent larvae from breathing properly while pupae are already affected in their breathing when the surface tension is reduced to 41 dynes/cm [55]. In the case of adults, emergence is already prevented when the surface tension is lowered to 38 dynes/cm [19]. As a result, a monomolecular film that loses efficacy over time, is able to control pupae and adults longer than larvae.

Both Aquatain applications reduced culicine larval densities although the effect of Aquatain application lasted for only five days after the second application. Culicine mosquitoes are known to be less affected by monomolecular films compared to anophelines. Reiter (1978) showed that, after a few failed attempts, culicine larvae could penetrate the lecithin film with their respiratory siphon unlike anopheline larvae that have different and shorter respiratory structures [16], [56]. In addition, culicines can thrive in conditions with low levels of dissolved oxygen which is evident by their presence in polluted water [27]. Culicine larvae were also less sensitive to the other two monomolecular products Arosurf MSF and Agnique MMF [22], [24], [26], [27]. Aquatain, however, reduced the number of culicine larvae in our experimental rice fields considerably.

Water turbidity had a negative effect on larval densities before Aquatain application. Turbidity reduces larval survival; however, in this study it might be because the larvae were flushed out while the plots were being flooded [57]. During irrigation, water was allowed to enter a plot from one end until water started to flow over the edge into the drainage canals at the other end of the field. The water was turbid due to its flowing in unlined, earthen irrigation canals. As a result, low larval densities and high turbidity were recorded together. A similar effect of high turbidity levels was also recorded for non-target organisms. The water surface temperature was found to have a positive effect on larval densities after both Aquatain applications. Higher larval densities were recorded at higher temperatures. A low water surface temperature (∼20°C) was recorded after either irrigation or rainfall. As no irrigation took place after Aquatain applications, the low temperatures in our rice fields were due to rainfall which has been shown to reduce survival of *An. gambiae* larvae. Reduced larval survival was considered to be due to direct impact of a rain drop or exhaustion of larval energy reserves from continuous diving in response to water turbulence [58].

During the two applications of Aquatain two doses were tested. The low dose (1 ml/m^2^) had almost no effect on larval densities. A contributing factor to this low efficacy against larvae may have been the hand-weeding carried out during that time (Figure 2). The amount of Aquatain in the rice paddies may have been reduced by the movement of workers in and out of the plots as Aquatain adsorbed onto their clothing. It is, however, noteworthy that even under these conditions Aquatain reduced anopheline adult emergence by 93%.

Knapsack or ULV sprayers have been used to apply other monomolecular films in rice paddies [18], [34]. Aquatain, however, could simply be poured at one location into the rice paddies. Aquatain film was visible and effective against larvae for 10 days after the second application (2 ml/m^2^). Application of Aquatain at intervals of 10–15 days is, therefore, necessary to effectively reduce mosquito densities. This is in agreement with the treatment cycle of Arosurf MSF, which was effective when applied every 14 days [34]. Lecithin, on the other hand, was shown to be effective for only two days under similar field conditions [18]. However, in less vegetated breeding sites the required dose of Aquatain might be lower and the re-application period longer because of a stronger effect on larval survival. This effect was evident in the field bioassays where no pupation was recorded in the Aquatain-treated buckets compared to 40% pupation in the control buckets.

Non-target organisms like biting flies, house flies, grasshoppers etc. that have terrestrial larvae or nymphs were caught in the emergence trap probably because they were enclosed while the emergence trap was placed at a new position or entered through a hole (repaired once detected) in the net or through the narrow gap (1–2 cm) between the emergence trap and water. Aquatain had no discernible negative effect on non-target organisms except for backswimmers (Notonectidae). Among the non-target organisms collected, broad shouldered water striders, backswimmers and water beetles are known to rely on the air-water interface for movement or respiration. In rice paddies, the presence of vegetation, both rice plants and algae, may have provided a substrate to broad shouldered water striders to hold on to, preventing them from drowning. Similar to mosquito larvae, the water beetles and backswimmers may have utilised the untreated water surface in between the algal mass to pick up the air bubble they require for respiration. However backswimmers are active predators and are known to detect their prey by the surface waves [59]. The velocity of the surface waves depends on the surface tension. As a result reduced surface tension of water might have affected their ability to forage for prey and therefore reduced their survival. Reiter (1978) observed mortality in (unidentified) adult Diptera and Ephemeroptera, one day after the lecithin and kerosene oil mix was sprayed [18]. However, generally no non-target effect was identified. Takahashi *et al*. (1984) in semi-field conditions showed that ISA-20E (0.25–1 ml/m^2^) caused mortality after a day in backswimmers, water boatmen, clam shrimp and water beetles but not on mayfly nymphs, chironomid larvae and copepods. The population level of back swimmer and water boatmen resurged to pre-treatment levels by the third day [28]. In our study the first sampling after Aquatain application was carried out after 5–6 days, which might explain why we did not identify any non-target effect. In a laboratory study, Hester *et al*. (1991) showed no adverse effect of Arosurf MSF (47 ml/m^2^) on snails, isopods, polychaetes, fiddler crabs, fresh water and grass shrimps, and long nose kill fish [33]. Other laboratory and field studies also reported no negative effects of monomolecular layers on non-target organisms and thus specifically targeted mosquito larvae [34], [37].

Our study demonstrated that Aquatain film had no adverse effects on the growth and development of rice plants in treatment paddies. The crop yield was similar in the control and treatment plots. This is consistent with the findings of Hester *et al*. (1989) In their study, Arosurf MSF (∼1 ml/m^2^) was applied from the top (except in mangroves). No plant injury or negative effect on the growth and development of aquatic plants, including rice, was observed [32].

Aquatain did not reduce the evaporation of water in the bioassays. Nitrogenous fertilizer was applied soon after the 15 rice seedlings were planted in the treatment tubs which accelerated the vegetative growth. Aquatain was applied when rice plants had clearly established in the tubs. By then, due to their height the rice plants covered the water surface and reduced the amount of sunlight reaching the water surface [10]. As a result, temperature of the water in the tubs may have been low resulting in low evaporation. Planting fewer rice seedlings (3 to 5) may have provided a better insight. By contrast, in the assay with tubs without soil and rice plants nearly 1.7 L of water was saved over a period of six days from a water surface area of 0.2 m^2^. Based on these results it can be estimated that under similar climatic conditions ∼34,400 liters (9087 gallons) of water can be saved from a one acre rice paddy over six days during the early stages of a rice crop when sunlight still reaches most of the water surface [10]. The implications of this for the farmer, who spends 8–12 % of the rice crop income on water needs, in terms of reduced water cost, needs to be further investigated.

A few limitations experienced during this study were (a) a pesticide (Titan, acetamiprid) had to be sprayed in six paddies (3 control, 3 treatment) two weeks before the first Aquatain application. As a result fewer larvae were caught after the first Aquatain application in both control and treatment paddies; (b) Due to logistic problems Aquatain was applied only at the second half of rice crop development. At this time of the rice crop, larval densities are known to be lower [10]; (c) frequent larval sampling (every 2 days) was required but not logistically possible to observe the effect of Aquatain on the late and the more susceptible stages of larvae.

One of the reasons why monomolecular films have not been extensively employed in mosquito control programmes is their sensitivity to wind [20]. Wind speeds higher than 12.9 km/h moved Arosurf MSF upwind [24]. When formulated with 2-ethyl butanol or 2 propanol Arosurf MSF was effective even with wind speeds up to 48.3 km/h. However, in this case the film was only persistent for three days [27]. Aquatain itself has been tested and is known to be resilient to wind speeds up to 41 km/h [43]. In our study, however, the maximum wind speed recorded was 5.2 km/h, five days after the first Aquatain application. Therefore, we could not yet establish how resilient Aquatain is to higher wind speeds in an irrigated rice setting.

In conclusion, Aquatain effectively and specifically controlled anopheline and culicine mosquitoes in rice paddies. It also showed potential in saving irrigation water under certain conditions. This makes it an ideal mosquito control tool for use in rice agro-ecosystems. Presently, adult mosquito control is the main malaria vector control strategy in Africa [60]. However, insecticide-treated bed-nets and indoor residual spraying do not provide protection outdoors. Besides, early night feeding behaviour, when people are still active, threatens the efficacy of tools that prevent mosquitoes biting at night [61]. There is also growing evidence of resistance developing in mosquitoes against insecticides used to treat bed nets and for indoor residual spraying [62]–[64]. An integrated approach is required to manage and mitigate these foreseen limitations of adult control. *Bacillus thuringiensis* var. *israelensis*, a microbial larvicide, complemented adult control and reduced malaria infections in highland areas [65]. Similarly, Aquatain, although not strictly a larvicide, can be employed in rice agro-settlements and considered an effective tool in the prevention of malaria transmission.
